# Red Sea Opisthobranchia 6: Phyllidiidae and their paradorid mimic: new species and new records (Heterobranchia, Nudibranchia, Doridina)

**DOI:** 10.3897/zookeys.1006.59732

**Published:** 2020-12-21

**Authors:** Nathalie Yonow

**Affiliations:** 1 Department of Biosciences, Swansea University, Singleton Park, Swansea SA2 8PP, Wales, United Kingdom Swansea University Swansea United Kingdom

**Keywords:** Biogeography, Discodorididae, endemism, *
Paradoris
*, *
Phyllidia
*, *
Phyllidiella
*, *
Phyllidiopsis
*, taxonomy

## Abstract

With the complexity of the family Phyllidiidae and the problems of identification in the Indo-West Pacific, the story of the Red Sea species continues to unfold. One new species and one new record are added to the Red Sea fauna, both belonging to the genus *Phyllidiella*. *Phyllidiella amphitrite***sp. nov.** is described from a single specimen from the northern Red Sea and clearly differs from all species of *Phyllidiella* in having pale yellow pigment on the tubercles. *Phyllidiella zeylanica* is newly recorded from the Red Sea with five specimen records and several photographed individuals; other than this, it has a western Indian Ocean distribution. *Phyllidia schupporum* was collected for the first time since its original description; because its distribution is now extended to the Persian Gulf, it is no longer considered endemic to the Red Sea. The distribution of the Red Sea endemic *Phyllidia dautzenbergi* is extended northwards to Hurghada, Egypt. A small specimen of the endemic *Phyllidiopsis sinaiensis* was found at 214–237 m depth just at the mouth of the Red Sea, which is a bathymetrical range extension from its previous shallow coral reef records. The identifications of other species are revisited. A new species of *Paradoris* is described as *Paradoris hypocrita***sp. nov.**, differing from the well-known but localised West Pacific *P. liturata* which also resembles a phyllidiid. This new species was recorded many years ago by published photographs, and it is relatively common in the Red Sea, evidenced by several specimens and additional photographs. It is described herein, and is considered a Red Sea endemic, differing from both the unnamed Indian Ocean species and the named Pacific species.

## Introduction

Species of the family Phyllidiidae Rafinesque, 1814 are commonly found throughout the tropical Indo-Pacific realm but two species are known from the Mediterranean and five from the tropical Atlantic Ocean. They belong to the Doridina but differ radically from most other members of the suborder (such as the Discodorididae Bergh, 1891) in not having dorsal circumanal gills, jaws, or a radula. The anus is still postero-dorsal in all genera except in the subgenus Fryeria Gray, 1853 where it is present ventrally at the very posterior of the body. Secondarily developed gills lie ventrally between the foot and mantle as a series of leaflets. Modification of the digestive tract is regarded as an advanced character in the nudibranchs which mostly have jaws and a radula (Wägele 1985).

Most Indo-Pacific phyllidiid species are characterised by a tough, rubbery, domed body, generally elongate oval, and variably covered in many types of tubercles and/or black lines. Species range in size from approximately 1 to 100 cm, and are generally colourful, but a few are pink, white, or beige with or without black markings. The lamellate rhinophores are orange, black, ochre, or pink and black. Ventrally, they have a foot which is smaller than the body, but its anterior end is sometimes species specific. The foot margin can be rounded, squared, dented, or notched. A bilobed part (termed ‘lips’ in this work) sits just below the mouth but above the foot margin. Above the mouth are oral tentacles which bear a groove on their outer sides but vary in shape between the genera: separate and digitiform, contiguous and triangular, or fused, with some specific variations. They may be coloured orange, yellow, pink, or grey, and tipped with black in some species.

This work describes three species of Phyllidiidae and the mimic belonging to the genus *Paradoris* Bergh, 1884 (family Discodorididae) from the Red Sea that were not included in the previous taxonomic publications (Yonow 1986, 1988, 1996). Species are illustrated with colour images of living specimens, and the literature relating to the species in question is included in the synonymies, focusing on the Red Sea, Arabian Sea, and the wider north–western Indian Ocean if relevant.

**Map 1.**
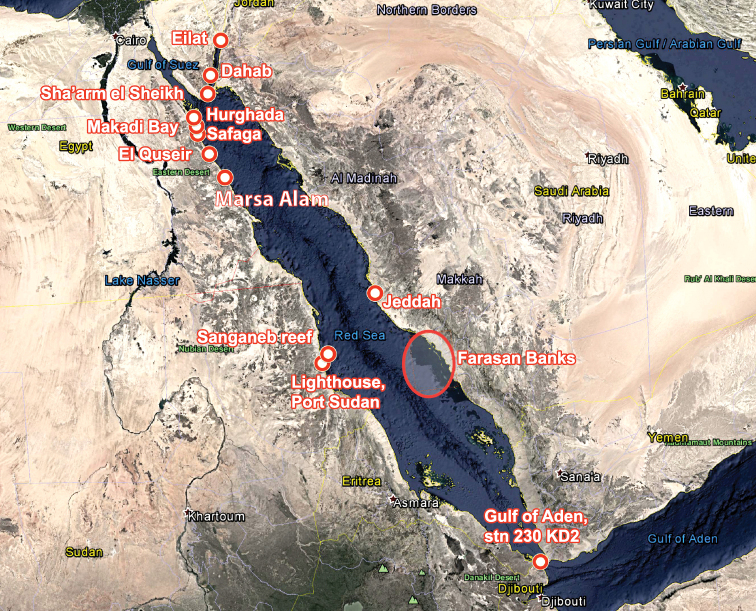
Map of the Red Sea showing all the collecting and photograph sites listed in this paper.

Fahrner and Schrödl (2000a) recognised eleven species from the Red Sea and synonymised several with their Indo-Pacific counterparts that were the most similar. Therefore they included *Phyllidia rosans* (Bergh, 1873), *Phyllidiella annulata* (Gray, 1853), *Phyllidiella elegans* Bergh, 1869, and *Phyllidia ocellata* Cuvier, 1804 into the Red Sea fauna, providing an endemism figure of 36% for the family Phyllidiidae. These identities are discussed herein and a check-list of the species belonging to the family Phyllidiidae recorded from the Red Sea to date is included. Additionally, Appendix 1 lists the remaining specimens in the author’s collections that refer to species previously described in this series, each with a brief diagnosis, remarks where relevant, and colour plates of the living animals of two species that were not available when they were originally described, *Phyllidiopsis monacha* (Yonow, 1986) and *Phyllidiopsis sinaiensis* (Yonow, 1988). Appendix 2 lists 25 specimens of the family Phyllidiidae recently collected from the Farasan Banks (see Map 1) which comprises only three of the most common species in the Red Sea, *Phyllidia multifaria* Yonow, 1986, *Phyllidia varicosa* Lamarck, 1801, and *Phyllidiella* ‘*pustulosa*’ (Cuvier, 1804).

## Materials and methods

The material described and listed in this paper were collected and/or photographed by several divers with one exception, the dredges of the deep-sea expeditions by RV ‘Meteor’. The fifth cruise of this research vessel took place in the Red Sea and the Gulf of Aden in February and March 1987, and among the opisthobranchs collected was a phyllidiid. A map illustrates the positions of the collecting localities of the specimens and photographs included in this paper, ranging from Israel, Sinai, northeast Egypt, Saudi Arabia, Sudan, and the Bab el Mandab (Gulf of Tadjourah) at the mouth of the Red Sea (Map 1). All the coordinates of the localities mentioned in text are provided in Table 1.

**Table 1.** Georeferenced collection and photographic localities, from north to south.

Main location	Dive site	Latitude / Longitude
Eilat		29°32'55.0"N, 34°57'19.1"E
Dahab		28°29'37.8"N, 34°31'05.1"E
	Moray Garden	
	Sha’ab Mahmoud/ Beacon Rock	
Sha’arm el Sheikh		27°55'59.7"N, 34°22'41.5"E
	Whale Bay	
	Thistlegorm	
	Ras Umm Sid	
	Near Gardens	
Hurghada		27°15'08.1"N, 33°50'55.3"E
	Sha’ab Dorfa	
	Abu Kafan	
	Small Gubal Island	
Makadi Bay		26°59'10.4"N, 33°55'04.0"E
Safaga		26°44'35.7"N, 33°56'42.5"E
El Quseir		26°06'03.4"N, 34°16'58.9"E
Abu Dabbab		25°20'11.9"N, 34°44'19.7"E
	Marsa Alam	
Jeddah		21°27'26.5"N, 39°08'35.3"E
	Obhur Creek	
Sanganeb reef		19°50'53.0"N, 37°27'27.7"E
Lighthouse, Port Sudan		19°36'06.1"N, 37°14'35.9"E
Farasan Banks		18°20' – 20°00'N, 40° – 41°20'E
Gulf of Aden, stn 230 KD2		12°43.50'N, 43°14.8'E

The collected material was not always measured, photographed, or relaxed before preservation, but as soon as the specimens were received, each one was examined under a Leica MZ APO microscope, and measurements, notes, and drawings were made. Some arrived preserved in formalin, others in various alcohols. In some cases, photographs were taken of the preserved specimen. After detailed descriptions of the dorsal and ventral sides were made, the digestive anatomy was examined by making a circular dorsal incision. Final drawings are a composite tracing of a series of printed photographs in combination with the annotated drawings. The features of the radulae and jaws of *Paradoris* specimens were analysed under a stereomicroscope and scanning electron microscope (CamScan Series II, JSM 6380).

In the Material section of each species and in the appendices, **specimen** refers to a collected animal which has been preserved, registered, and will be lodged in the Senckenberg Museum, Frankfurt**(SMF)** (or Naturalis Biodiversity Center, Leiden, the Netherlands for the Farasan Banks collection), while **individual** refers to an animal which was photographed, sometimes measured alive, but not collected. Colour slides and digital images of the specimens and individuals included in this paper will also be deposited in the SMF with their specimens and SEM stubs.

## Species accounts

### Phyllidiidae Rafinesque, 1814

#### Phyllidiella amphitrite sp. nov.

Taxon classificationAnimaliaNudibranchiaPhyllidiidae

E8DA8F4A-93C1-5F1A-9C85-A23EE4C76E34

http://zoobank.org/B451CAFE-B222-4A31-B891-F9A932594EA8

**Plate 1.**
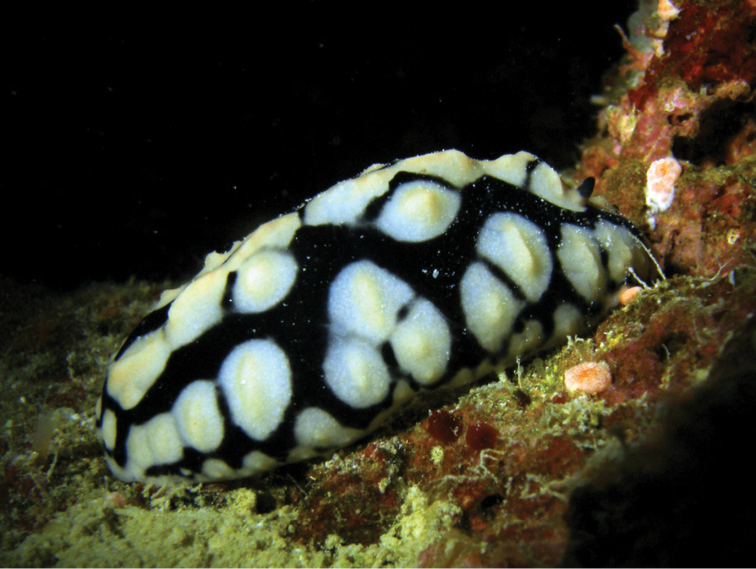
*Phyllidiella amphitrite* sp. nov., holotype SMF 360585, Hurghada, Egypt, 09 August 2009 (photograph S. Kahlbrock).

**Figure 1.**
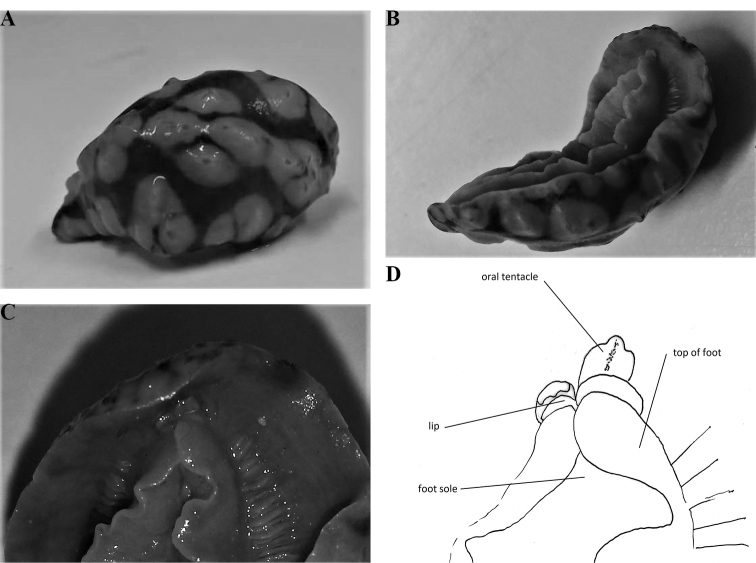
*Phyllidiella amphitrite* sp. nov., holotype SMF 360585 **A** dorsal view **B** ventral view **C** detail of oral tentacles and anterior foot margin **D** drawing of oral tentacles, ‘lips’, and anterior foot margin.

**Figure 2.**
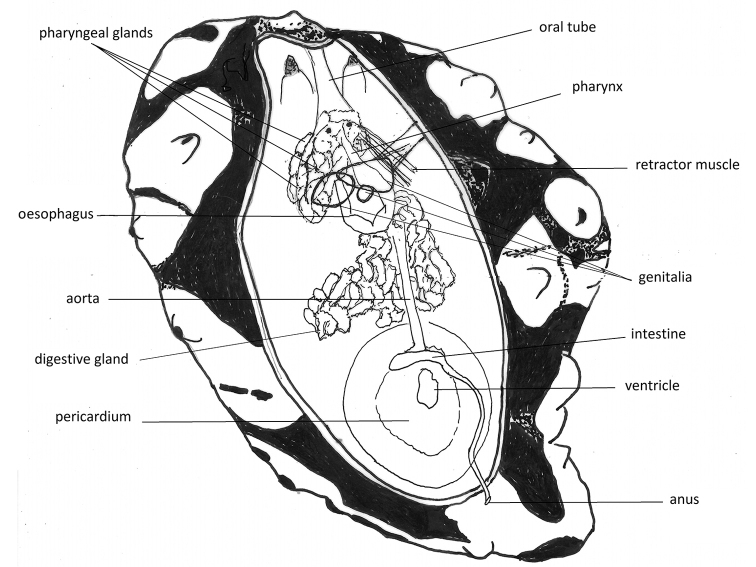
*Phyllidiella amphitrite* sp. nov., holotype SMF 360585, drawing of the digestive anatomy and the genitalia (the latter in heavier ink).

Plate 1 ; Figures 1 , 2

##### Material.

***Holotype*.**SMF 360585. Near Hurghada, Egypt, 09 August 2009, one specimen 28 × 15 mm, bent (pres., alcohol), leg. and photograph S. Kahlbrock.

##### Diagnosis.

Relatively smooth phyllidiid with pale yellow crests and cones (instead of rounded tubercles). White areas granulate, black areas smooth. Sub-margin with single series of yellow and white patches and small crests and pointed tubercles, margin smooth and pale. Rhinophores black, extending from pale yellow raised sheaths. Very distinctive oral tentacles, trilobed with a median ridge (not digitiform as those of other species of *Phyllidiella*). Dorsal anus.

##### Description.

The photograph of the living specimen depicts an animal which vaguely resembles a smudged *Phyllidiella* ‘*pustulosa*’ with yellow pigment on the top of its crests, which are loosely arranged in groups with black lines around them (Plate 1). These tuberculate areas comprise a large white area and the tubercles arise very sharply as crests or cones, both pale yellow. These crests are low, and those around the margin are either low cones or rounded on top. There are six of these elongated crests forming a ridge along the midline, grouped into three polygonal areas with two single ones situated behind the anus. The rhinophores are located on the anterior sides of the first cluster, and issue from raised sheaths which are faintly yellow; the rhinophores are associated with rhino-tubercles. The visible part of the right rhinophore in the photograph is black and densely lamellated, rounded at the tip. The individual marginal tubercles are very small and nipple-like with a large creamy white base. They are present as a single series along the submargin and the margin is smooth and pale.

The preserved specimen is curved ventrally but everything is clearly visible (Fig. 1A). The dorsal crests and cones are still present in the specimen and appear ‘dirty’ where they were coloured yellow. The anus is located on the posterior edge of the third tubercular cluster. The right rhinophore was removed and bears 17 lamellae, the lowest four of which are white, and a rounded distal tip. Ventrally, there is no black showing through the hyponotum, nor are there any black markings on the gill leaflets or oral tentacles in the preserved specimen. The foot sole has no black line. The oral tentacles, gill leaflets, and gonopore are all flesh-coloured (Fig. 1B, C). The oral tentacles are trilobed with a median ridge: the right one (specimen viewed ventrally) is upstanding and the left one is folded over (Fig. 1C, D). The foot is folded longitudinally (Fig. 1B, C, D).

Anatomically, the dissection of the single specimen confirms placement in the genus *Phyllidiella* (see Fig. 2). The internal organs were covered by a dark visceral envelope and beneath this was a smaller envelope anteriorly covering the pharynx, pharyngeal bulb, oral glands, and nervous system. When this was removed, a large mass of large leaf-like oral glands covered the pharynx and pharyngeal bulb. The first is long and muscular, forming a large loop. Two strong muscles attach the elongated and bent pharyngeal bulb to the body wall. The bursa copulatrix is a solid sphere with a reddish patch on the ventral-most side.

##### Remarks.

The internal anatomy of this new species clearly places it in *Phyllidiella*: the visceral envelope is black, the pharyngeal bulb is elongate and folded, the pharynx is thick and muscular but becomes tubular, and there are leaf-like glands overlying the pharynx and the bulb. However, there are no other known species of *Phyllidiella* with yellow pigmentation or with such unusual oral tentacles. Despite these two differences, it is described as a new member of *Phyllidiella* due to similarities in the digestive system. *Phyllidiella ‘pustulosa*’ is one of the most common species in the Red Sea and Indo-West Pacific, but there are no records of it having yellow tubercles or crests on the tubercles.

**Plate 2.**
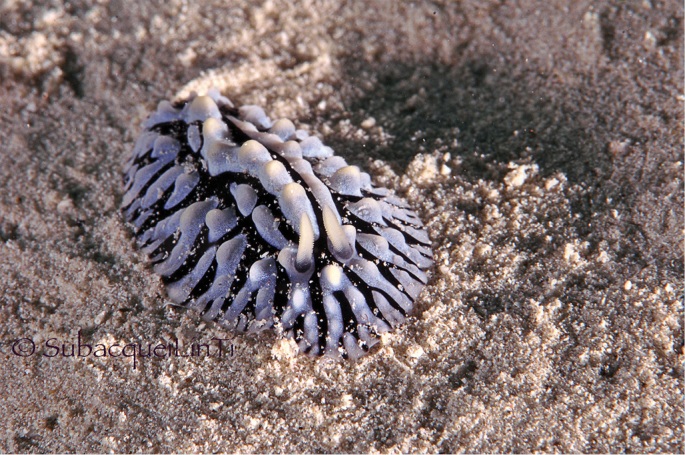
*Phyllidia varicosa*, not collected, very pale individual, Makadi Bay, Egypt, 2013 (photograph Hsini Lin).

**Plate 3.**
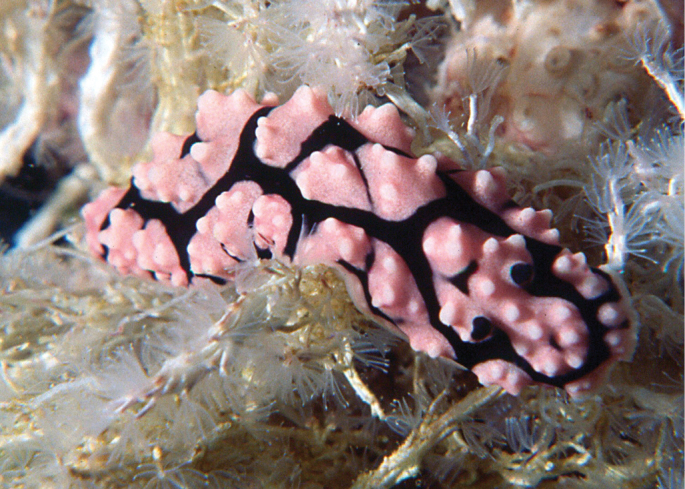
Phyllidiella ‘pustulosa’, not collected, with pointed tubercles, Obhur Creek, Jeddah, Saudi Arabia, 1980’s (photograph W. Pridgen).

*Phyllidiella ‘pustulosa*’ is always pink, green, or white underwater and in photographs, possibly depending on the lighting utilised; no species of *Phyllidiella* has any yellow pigmentation. There are, however, instances of very pale yellow markings in other genera, which may lead to misidentifications, e.g., *Phyllidiella* sp. in Gosliner et al. (2008: 295) which is in fact *Phyllidia elegans*: the pattern of black and tubercles is typical, and a yellow tinge is clearly visible on the rhinophores. For comparison, a photograph of a very pale *Phyllidia varicosa* is illustrated in Plate 2, and another is available on Sea Slug Forum (Adams 2003). There are specimens in the Red Sea of *P.* ‘*pustulosa*’ with a more pointed appearance (Plate 3) which may eventually also be identified as a different species; but, it must be noted that these spikier variations also occur in other phyllidiid species, e.g., *Phyllidia multifaria* (Yonow, 1986: 1410, fig. 11i; Yonow 1988: 149, pl. 7).

While *P. amphitrite* looks like an aberrant form of *P.* ‘*pustulosa*’, the differences are enough to warrant specific separation for now, especially following the recent molecular work on the species complex in the western Pacific (Stoffels et al. 2016; Bogdanov 2020; see Remarks for *P.* ‘*pustulosa*’ in Appendix 1). No other phyllidiid has trilobed oral tentacles, a character which needs further examination when more specimens are collected. Species of the *Phyllidia pustulosa* complex have triangular oral tentacles with a lateral groove and are tipped in black. With black rhinophores and a mass of leaf-like oral glands, *P. amphitrite* clearly does not belong to *Phyllidia* or *Phyllidiopsis*, and it is placed in *Phyllidiella* as the most parsimonious choice.

##### Etymology.

The name was chosen for the wife of the ruler of the sea, Poseidon, in Greek mythology. She was called *Ἀμφιτρίτη*, Amphitrite.

#### Phyllidiella zeylanica

Taxon classificationAnimaliaNudibranchiaPhyllidiidae

(Kelaart, 1858)

EECE00D4-184D-507A-997F-53908658F1EE

Plates 4–8 ; Figures 3–6

Phyllidia zeylanicus Kelaart, 1858: 120 (Sri Lanka).
Phyllidiella zeylanica : – Yonow 1996: 502, fig. 10A–G (Maldives, Seychelles, Thailand); Yonow et al. 2002: 868, fig. 19a (Chagos); Domínguez et al. 2007: 105, fig. 12 (Papua New Guinea); Yonow 2012: 71, pls 72, 73 (Seychelles. Maldives, Sri Lanka) and references therein.

##### Material.

**Egypt** – Hurghada. Sept 2009, one specimen 10 × 5 mm (pres., alcohol), leg. and photograph S. Kahlbrock; Sha’ab Dorfa, 07 Sept 2010, 14 m depth, one specimen 10 × 5 mm (pres., alcohol), leg. and photograph S. Kahlbrock; 2014, one specimen 13 × 6 mm (pres., formaldehyde), leg. S. Kahlbrock; April 2015, one specimen 9 × 5 mm (pres., alcohol), leg. S. Kahlbrock; Abu Kafan, 14 July 2015, 7 m depth, one specimen 10.5 × 4 mm (pres., alcohol), leg. and photographs S. Kahlbrock (SK #13).

##### Photographic records.

**Egypt** – Hurghada, 16 July and 15 Aug 2010, three individuals, photographs S. Kahlbrock; Dahab, 2017, photographs of one individual, C. von Mach (H. Blatterer, Vienna, pers. comm.).

##### Description.

**Plates 4–8.**
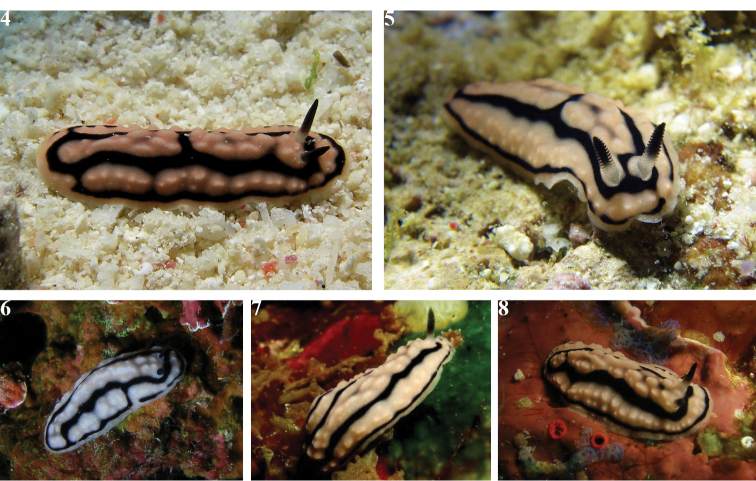
*Phyllidiella zeylanica*, variations in dorsal pattern **4** Hurghada, April 2009 **5** Abu Kafan, 14 July 2015, SK #13 **6–8** three individuals not collected, near Hurghada (all photographs S. Kahlbrock).

**Figures 3–6.**
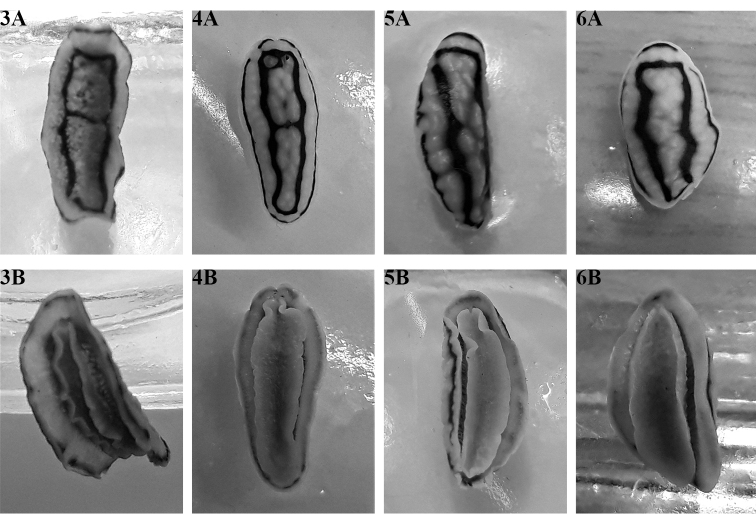
*Phyllidiella zeylanica*, four specimens. Upper row (**A**) showing the dorsal pattern and lower row (**B**) illustrating their ventral sides **3** Hurghada, April 2009, S. Kahlbrock **4** Abu Kafan, 14 July 2015, S. Kahlbrock SK #13 **5** near Hurghada, 2014, S. Kahlbrock **6** Hurghada, April 2015, S. Kahlbrock.

These five specimens and the additional photographs all bear a single dorsal black band enclosing both the rhinophores and the anal orifice (Plates 4–8, Figs 3A–6A); it is not quite complete in one specimen and bears a transverse mark in three specimens (Plates 4, 5, Figs 3A, 4A). Critically, in all five specimens, the anterior section of this black band is squared, a feature unique to this species. All specimens bear a second thin black line submarginally and faint black markings within the central black ring. The individual tubercles in the central black ‘square’ and the multiple tubercles in the wide marginal pink band are as described previously: the pink areas are tuberculate with some faint or distinct black markings between them. Between the two black rings is a double or triple row of tubercles, which appears to be another diagnostic character of *P. zeylanica*. The thin mantle margin is pink. The rhinophores are long and straight in all photographs, black with a white stalk and few white lower lamellae. Photographs of three individuals are also similar and clearly identifiable; the one with slightly higher and more defined tubercles is probably larger than the others (Plate 8), virtually identical to the specimen illustrated from the Maldives (Yonow 2012: pl. 73) that measured 38 mm in length.

None of the preserved specimens were relaxed before preservation, but they are moderately flat with the margins slightly curled (as reported previously for preserved specimens) and their rhinophores are all retracted. Of the photographic series of living specimens, SK #13 has a few that are focused on the rhinophores, and there are 12–14 lamellae on each clavus with the lower three or four lamellae being white. This lower white portion is visible on all photographs of all animals even if they are not sharp enough to count the individual lamellae. Ventrally, the foot sole has no black line nor are there any other markings on it or on the hyponotum (Figs 3B, 4B, 5B, 6B). In four specimens the anterior foot margin is notched and the margin and ‘lips’ are separated with the triangular oral tentacles set at an angle. In one less relaxed specimen, the ‘lips’ and margin are contracted around the mouth. In three specimens (Figs 3B, 4B, 5B), black pigment is visible on the oral tentacles.

##### Remarks.

A careful search of all photographic records in the author’s archives from Pam Kemp, Woody Pridgen, and Jürgen Kuchinke who were in Saudi Arabia and diving during the 1980s revealed no photographs of *Phyllidiella zeylanica*; size is presumably not the issue as they all had photographs of *Phyllidia dautzenbergi* Vayssière, 1912 (< 20 mm alive), which is similarly small. Is it reasonable to conclude that *Phyllidiella zeylanica* is a recent migrant? Given that there is no previous photographic evidence of this species in the Red Sea, in this work, it is considered a recent introduction from the Indian Ocean, where it is frequently recorded. Some authors (e.g., Brunckhorst 1993, Gosliner et al. 2008) have a different view on the identity of *P. zeylanica* based on Pacific specimens, but to date, having examined hundreds of specimens of species of phyllidiids from both the Indian Ocean and the West Pacific, this consistent colour pattern bears no resemblance to some specimens in the author’s collection from the Indian Ocean, currently unidentified, whose dorsal patterns match those illustrated by Brunckhorst (1993) and Gosliner et al. (2008) identified as *P. zeylanica*. Given these external morphological differences, these are not simply much larger specimens of *P. zeylanica*. Domínguez et al. (2007) recorded *P. zeylanica* from Papua New Guinea which has the same dorsal pattern, and anterior foot with prominent ‘lips’ and triangular tentacles so the species is known to occur in the western Pacific. However, neither Domínguez et al. (2007) nor Brunckhorst (1993) described the black tips on the oral tentacles for *P. zeylanica* as they did for ‘*P. pustulosa*’.

#### Phyllidia schupporum

Taxon classificationAnimaliaNudibranchiaPhyllidiidae

Fahrner & Schrödl, 2000

41FDA3B1-CD16-5A9E-99E5-51C773AA8A4C

Plates 9–14 ; Figures 7 , 8

Phyllidia schupporum Fahrner & Schrödl, 2000b: 5–60, figs 1–4 (Dahab, Gulf of Eilat); Debelius and Kuiter 2007: 267; Yonow 2008: 217, three figs incl. ventral anterior of holotype; Gosliner et al. 2008: 289.
Phyllidia (Fryeria) rueppelii : – Nithyanandan 2012: 4, fig. 6 (Kuwait). non P. (F.) rueppelii (Bergh, 1869).

##### Material.

**Egypt** – Hurghada, Sha’ab Dorfa, 7 Sept 2010, one specimen 32 × 21 mm pres., alcohol, 14 m depth, leg. and photographs S. Kahlbrock.

##### Photographic records.

**Egypt** – Caves, Ras Umm Sid, Sha’arm el Sheikh, 19 Nov 2007, one individual, photograph H. Blatterer; El Quseir, 2007, one individual, photograph H. Blatterer; Dahab, 2008, two individuals together, photographs H. Blatterer; Sha’ab Mahmoud/Beacon Rock, Dahab, 5 May 2010, 8 June 2010, 15 Apr 2012, photographs S. Kahlbrock; Hurghada, 14 May 2012, photograph of one individual, S. Kahlbrock; “SS Thistlegorm,” Sha’arm el Sheikh, 9 Oct 2012, 17 m depth, photograph S. Kahlbrock; House Reef of Rima Life Resort, Makadi Bay, 9 August 2014, 8 m depth, one individual ~ 30 mm, photograph Hsini Lin; Dahab, 19 July 2015, one individual, photograph H. Blatterer. **Israel** – Eilat. 16 Apr 2008, photographs of one individual, B. and S. Koretz (also published in Gosliner et al. 2008: 289); 4 September 2015, photographs of one individual, R. Amar.

##### Description.

**Plates 9–14.**
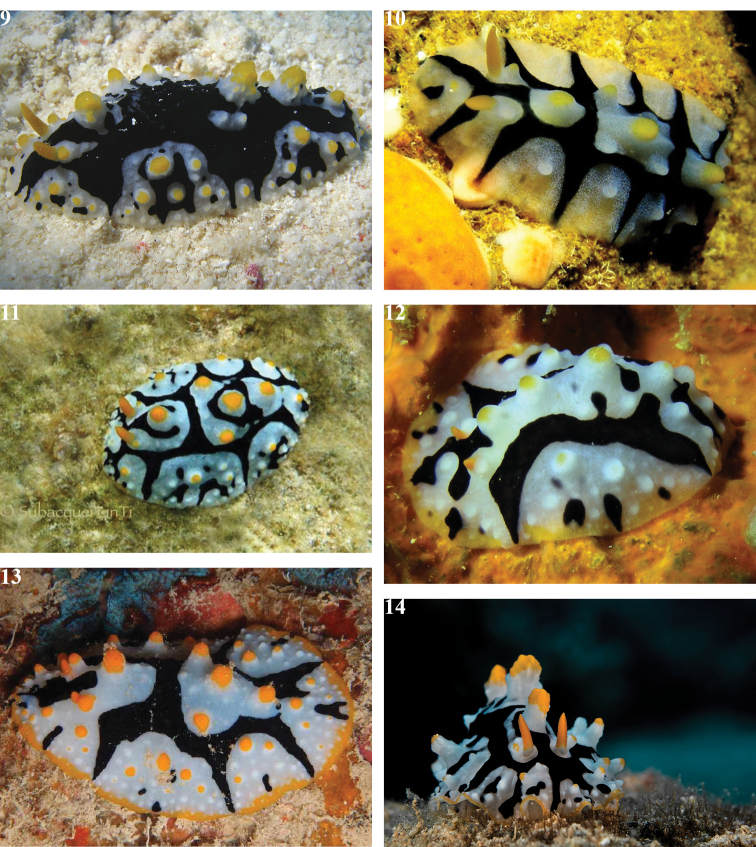
*Phyllidia schupporum*, specimen and individuals illustrating variations **9** dorsal view of living specimen, Sha’ab Dorfa, 7 Sept 2010 (photograph S. Kahlbrock) **10** Sha’arm el Sheikh, 15 Apr 2012 (photograph S. Kahlbrock) **11** Makadi Bay, 9 Aug 2014 (photograph Hsini Lin) **12** Sha’arm el Sheikh, Oct 2012 (photograph S. Kahlbrock) **13** Sha’ab Mahmoud/Beacon Rock, 9 Oct 2012 (photograph S. Kahlbrock) **14** Eilat, Israel, 4 Sept 2015 (photograph R. Amar).

**Figure 7.**
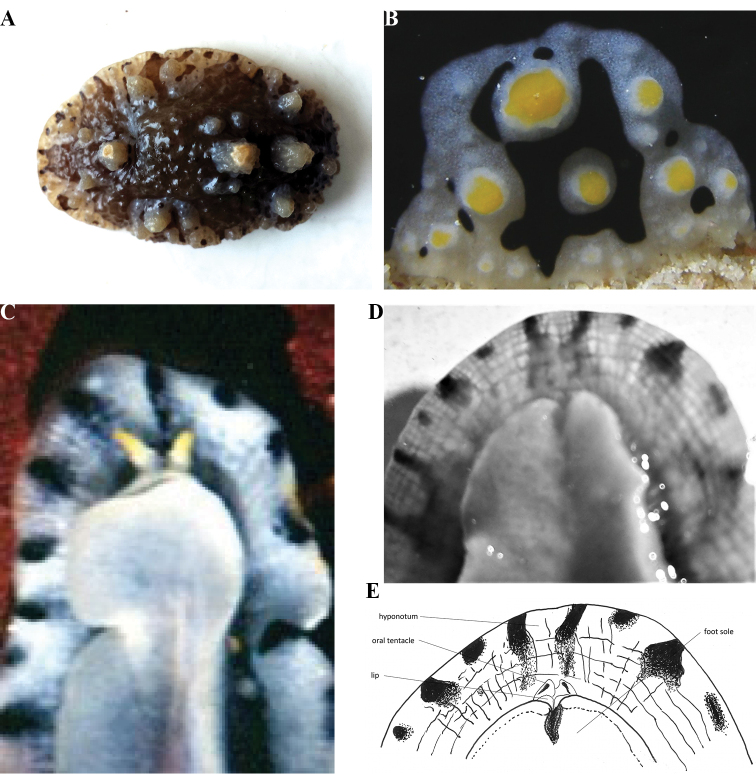
*Phyllidia schupporum***A** dorsal view of preserved specimen (same orientation as living animal in Plate 9 with head to the left) **B** detail of lateral tubercles and margin (lower) of specimen (enlarged from photograph of the living specimen **C** ventral view of holotype (M. Schrödl) **D, E** oral tentacles, ‘lips’ and anterior foot margin of specimen.

The single large (32 mm) preserved specimen is mostly black, which forms deep scallops around the mantle sides with three elongations on the left and four irregular ones on the right, almost reaching the mantle margin, and a small one on the posterior margin (Plate 9). These semi-circular areas formed by the black scalloped pattern are white but contain some black patches and bright yellow tips on both the large and small tubercles. The white areas are almost translucent and full of white granules; even the tiniest tubercles have granulated white pigmentation. The large tubercles in each area are white, topped with yellow, and the bases of the pigmented tubercles are more opaque white as well as more granular than the others, which makes them appear ocellated (Fig. 7B). These white semi-circles are pustulate, as are the yellow parts of the largest tubercles in these areas and elsewhere on the dorsum. There is no coloured edge remaining on the margin in this specimen, but all photographs of living animals show orange spots and/or lines on the mantle margin (Plates 9–14) and three display almost complete yellow margins (Plates 12–14).

The central black area in life bears a few barely discernible pustules, visible only at great magnification. The four large central tubercles are pustulose and irregular. There is one large tubercle just behind the rhinophores followed by two more. These three tubercles are the largest and rugose, with the basal pustules faintly tipped in yellow pigment which deepens towards tips. The white anal papilla is located just behind the third dorsal tubercle and located in a white area; the anal opening is surrounded by tiny black spots. There is one smaller white and yellow pustulose tubercle behind the anal papilla (far right on Plates 9, 11, 13). The deep yellow rhinophores are almost parallel sided with a short tip (Plates 9, 11, 14). They are each set in a tapering white patch with a yellow rhino-tubercle just behind and slightly displaced laterally. The rhinophores have 16–20 lamellae (counted from photographs).

The preserved specimen is black and white (Fig. 7A). Both the black and white areas are pimpled with small pustules, which are more obvious on the white areas. The tubercles are very unusual for species of *Phyllidia* and in preservative resemble the tubercles of *Dendrodoris tuberculosa* (Quoy & Gaimard, 1832). They are composed of ridges and incomplete rings of smaller pustules, some even tipped with yellow pigment, and very clearly visible in a large living animal (Plates 9, 14). The rhinophores are retracted and the anus is a puckered hole placed after the third tubercle and clearly visible.

Ventrally, the spicules of the hyponotum are arranged in a distinctly hatched pattern, and the black pigment shows through from the dorsal side, darkest around the margin (Fig. 7C–E). The gill leaflets are grey. The uniformly grey foot sole is oval with no black midline and notched anteriorly (Fig. 7D, E) in the preserved specimen but not in the living specimen (Fig. 7C). The anterior margin is rounded, the ‘lips’ are prominent, and the retracted oral tentacles are conical structures with an obvious deep groove on each side, similar to those depicted in the photograph of the living holotype (Fig. 7C, courtesy of M Schrödl).

**Figure 8.**
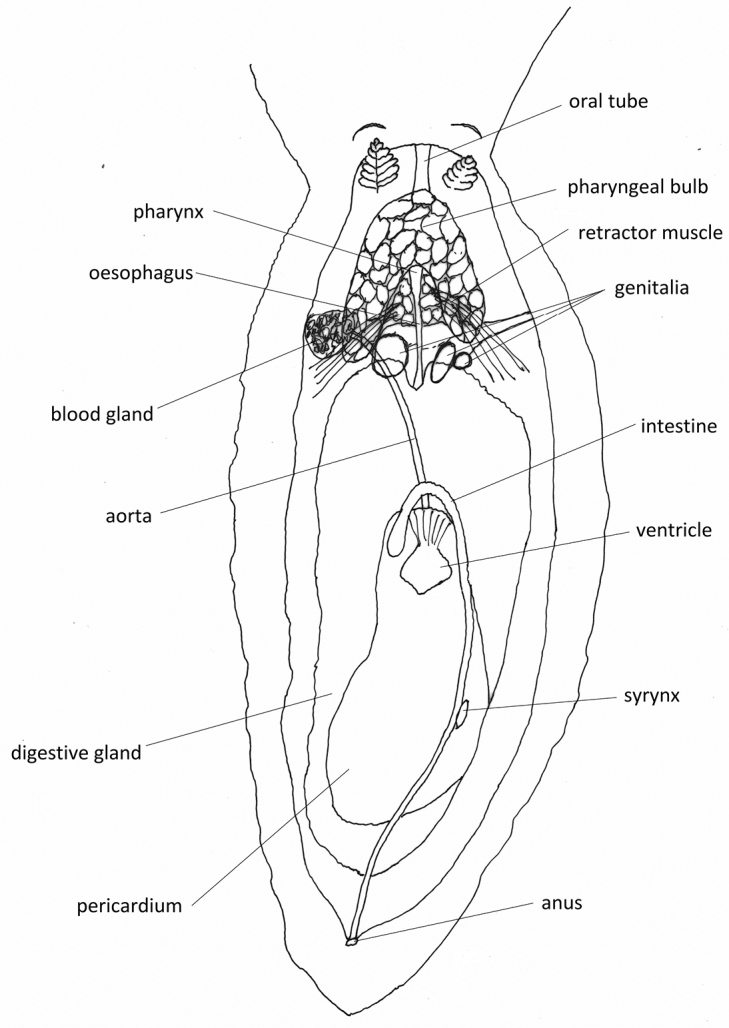
*Phyllidia schupporum*, drawing of the digestive anatomy, also showing the genitalia (in heavier ink).

A dorsal incision to remove the very thick notum revealed a digestive system (Fig. 8) similar of that of other species of the genus excluding the *multituberculata* Boettger, 1918 complex [e.g., *P. varicosa*, *P. alyta* (Yonow, 1996: figs 7–9), *P. coelestis* Bergh, 1905 (Yonow 2011: figs 14, 15), *P. koehleri* Perrone, 2000 (Yonow 2012: fig 19)] and matches the drawing by Fahrner and Schrödl (2000b: fig. 2). The pharyngeal bulb is creamy yellow and bears an upside-down U-shaped concavity on the dorso-posterior side; this is where the retractor muscles attachments and the small pharynx originate. The creamy-white blood gland lies in or over this concavity. The bursa copulatrix (left sphere) is a solid yellowish colour.

##### Remarks.

The external details of this specimen described in this work clearly fit those given for the holotype by Fahrner and Schrödl (2000b) despite the preponderance of black and the lack of orange- or yellow-tipped oral tentacles: as no orange/yellow pigment remains on the dorsum of the preserved specimen it is not surprising that the oral tentacles have lost their pigmentation. The species is distinctive, recognised in Yonow (2008) from photographs (see also references listed in the synonymy). Whilst this specimen is much darker than both the holotype and the available photographs, it does bear the diagnostic characters of three large, tall, spiculose, and pustulose orange/yellow-tipped tubercles along the dorsal midline, the presences of a smaller one posterior to the anus, two longitudinal black lines with at least one transverse band, an anterior black Y-shaped mark originating between the rhinophores and extending to the anterior margin, three or four white semi-circular areas on the margin on each side, orange/yellow rhinophores, and no black line on the sole of the foot.

The individuals photographed (Plates 10, 11, 13) are most similar to the holotype, but the black markings are thinner. In four individuals, there are three complete transverse lines (Plates 10, 11, 13, 14) which match the incomplete bands of the holotype.

Internally, the digestive system is as described and illustrated in Fahrner and Schrödl (2000b: figs 2, 3) but it no longer retains any bright orange-red colour that they described in the freshly collected animal.

One additional character for *P. schupporum* observed from this material should be added to its diagnosis: there is an orange or yellow border present on the mantle margin which is usually very patchy: it can be almost absent or almost entire with only small breaks. It is in fact present as one patch on the coloured illustration of the holotype (Fahrner and Schrödl 2000b: fig. 1) just ‘above’ the tip of the right rhinophore. Of the records presented here, all images examined at high magnification also show at least small marginal patches of orange or yellow. Therefore, all records of Phyllidia (Fryeria) rueppelii (Bergh, 1869) from the Red Sea, Persian Gulf, and Gulf of Oman must now be re-examined considering this new observation. The illustrated individual from Kuwait (Nithyanandan 2012: 5, fig. 6) clearly belongs to *P. schupporum* and not to P. (F.) rueppelii due to the three high spiculose and tuberculate central tubercles and the dorsal black pattern, indicating that *P. schupporum* may not be endemic to the Red Sea. Individuals depicted in several photographs examined from the United Arab Emirates can be attributed to *P. schupporum* (Carole Harris, Sydney, Australia, pers. comm.). There are transverse bands present in some of her images and there are extensions to the sides forming white semi-circular areas as in P. (F.) rueppelii. Apart from P. (F.) rueppelii, there are no other species in the Red Sea which have an orange or yellow margin; however, the black pattern is different in the two species. Phyllidia (F.) rueppelii has three rows of central tubercles with a more linear black pattern, and it has a ventral anus.

The individuals and holotype with less black are similar to the Indo-West Pacific *Phyllidia exquisita* Brunckhorst, 1993, also noted by Fahrner and Schrödl (2000b) but the Red Sea *P. schupporum* differs externally, having only two (instead of three or four) curved black longitudinal lines extending from the fronto-lateral mantle margins to the posterio-lateral margins.

*Phyllidia schupporum* is very similar with its light and dark variations to the images of a species identified as *P. exquisita* from Hawaii, which is probably a new species (Pittman and Fiene 1999). It is remotely possible that the Red Sea species and the Hawaiian ones are the same, as they resemble each other externally including the very dark variants. However, given their very disjunct localities and that both regions have high levels of endemism, the same identity is unlikely but further collections will eventually resolve this issue.

*Phyllidia schupporum* is a rare species in the Red Sea, with only two known specimens and several photographed individuals in the last forty years. It was not present in the more recent collections in the southern half of the Red Sea (e.g., Sanganeb 1991 by T. Paulus; Farasan banks 2017 by KAUST) but was recorded as early as the 1980s (single photograph by Pam Kemp in Yonow (2008) from the Jeddah area, central Red Sea). There are photographs on the internet from the northern Red Sea which have been variously identified as *F. rueppelii*, P. cf. exquisita, and *P. schupporum*.

### Discodorididae Bergh, 1891

#### *Paradoris* Bergh, 1884

The genus *Paradoris* is a small one and the number of species it contains ranges from eleven (Dayrat 2006) to up to 20 in WoRMS, but WoRMS still includes species that were synonymised by that 2006 revision, and there are additionally a few undescribed species that await formal description. The genus is distributed worldwide, from the Mediterranean to both sides of the Atlantic, both sides of the Pacific Ocean, the Indian Ocean, and the Red Sea. Their external colourations and morphologies vary widely, and one named species mimics the family Phyllidiidae: *Paradoris liturata* (Bergh, 1905), recorded from Indonesia and Papua New Guinea but not from the Indian Ocean. In this work, a second mimic is described based on five specimens. It is included in this paper due to its resemblance to the genus *Phyllidiella* and has been tentatively identified in the literature as Paradoris cf. liturata (Rudman 2007a) or as *P. liturata* (Debelius and Kuiter 2007: 242, right photograph only, from the Red Sea).

##### Paradoris hypocrita sp. nov.

Taxon classificationAnimaliaNudibranchiaDiscodorididae

714BE3B3-DC63-5B99-A07A-482FC7B33BAE

http://zoobank.org/6CA445F8-9162-4F8F-86D8-9A7580F879D4

Plates 15–18 ; Figures 9 , 10 , 11 , 12

Paradoris liturata : – Debelius and Kuiter 2007: 242, right photo only (Red Sea; non P. liturata Bergh).
Aldisa sp. 2 Yonow 2008: 156 (Red Sea).

###### Material.

***Holotype*.**SMF 360586. Hurghada, Egypt, Sept/Oct 1995, one specimen 10 × 5 mm pres., leg. A. Valdés and E. Mollo (HU–08).

***Paratypes*.**SMF 360587. Whale Bay, Sha'arm el Sheikh, Egypt, May 1980, 10–15 m depth, one specimen 8 × 6 mm pres. curled, leg. and photographs B.E. Picton (BEP/RS3); SMF 360588. near Hurghada, Egypt, 22 Feb 2011, one specimen 15 × 9 mm pres. curled, leg. S. Kahlbrock (SEM of jaws and radula); SMF 360589. Near Hurghada, Egypt, 2012, two specimens 15 × 10 mm (A; penis extruded) and 12 × 10 mm (B; SEM of jaws and radula) pres. curled, leg. S. Kahlbrock.

###### Photographic material.

**Egypt** – El Quseir, 2007, photograph of one individual, H. Blatterer; near Hurghada, 14 Jul 2010, photographs of one individual, S. Kahlbrock; near Hurghada, 09 Sept 2010, photographs of one individual, S. Kahlbrock; Abu Dabbab, Marsa Alam, 28 Jul 2014, 24 m depth, photographs of one individual 30 mm, Hsini Lin (LIN_0805); Abu Dabbab, Marsa Alam, 15 Apr 2015, 23 m depth, photographs of one individual 20 mm, Hsini Lin (LIN_3209); Abu Dabbab, Marsa Alam, 2 Aug 2018, 24 m depth, photographs of one individual 30 mm, Hsini Lin (LIN–P8020094); Moray Garden, Dahab, 2019, photograph of one individual, H. Blatterer. **Israel** – Eilat, 2014, 31 July 2015, 13 May 2020, photographs of three individuals, R. Amar.

###### Diagnosis.

Body elongate-oval with a distinct dorsal hump, wide mantle skirt. Dorsum pink, granular, with paler to white nodules, and black lines. Black pattern as four or five paired polygons; first pair around rhinophores with one or two lines extending to frontal margin. Dorsal polygons have short lines extending over skirt to margins. One polygon in front of the gills and one around the gills. Rhinophores black with translucent white stalk; rims of pockets raised, translucent pink, very thin, with an irregular margin. Six gills tri-pinnate, translucent white; pocket large with raised pink rim.

###### Description.

**Plates 15–18.**
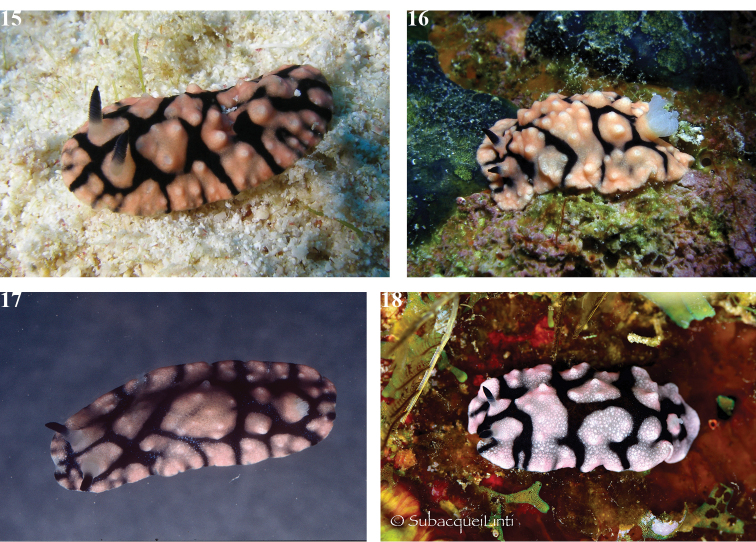
*Paradoris hypocrita* sp. nov., specimen and individuals illustrating constant patterns **15** Hurghada, 09 Sept 2010 (S. Kahlbrock) **16** Hurghada, 14 July 2010 (S. Kahlbrock) **17** paratype SMF 360587, Sha'arm el Sheikh, May 1980 (B.E. Picton) **18** Marsa Alam, 28 July 2014, photograph of 30 mm individual (Hsini Lin – LIN_0805).

**Figure 9.**
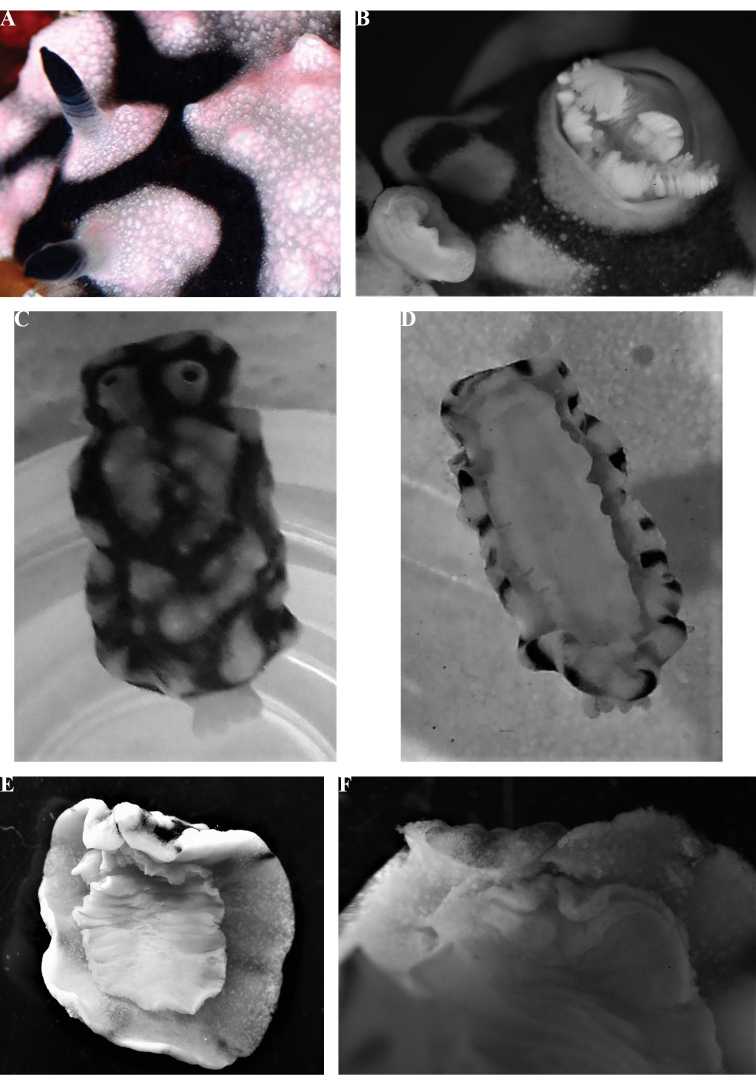
*Paradoris hypocrita* sp. nov. **A** detail of the granulation of the pink areas and the tubercles (photograph only, Hsini Lin LIN-0805) **B** gills partially extended, preserved paratype SMF 360587 **C** dorsal view showing extended gills, preserved holotype SMF 360586 **D** ventral view of preserved holotype SMF 360586 **E** ventral view of specimen A SMF 360589 with penis extruded **F** anterior bilabiate foot margin of specimen B SMF 360589.

The shape of the species is elongate oval, usually with an angular frontal margin. There is a central dorsal hump and a broad mantle skirt. The black markings are smooth, loosely paired in a series of four or five polygons, with a larger central one just in front of the gills. Each rhinophore and the gills are located within a polygon (Plates 15–18). The rhinophores are long, translucent white at the base and the lamellate clavus is black with a distinct squared tip that is angled. In three photographs of two living individuals, there are 17 lamellae in each of the four rhinophores that can be counted. The gill pocket is large when the gills are extended, with an upstanding pink rim; its margin appears irregular. The six tripinnate gills are translucent white and the edges appear denser white (Plates 16, 17). The pink areas are granular and covered in white tubercles that are also granular. An enlarged detail from one photograph shows that the granules vary in both size and density (Fig. 9A, from Plate 18).

The five preserved specimens (in alcohol or in formaldehyde) are all pale pink with approximately paired, rounded, polygonal, black markings (Fig. 9C). The black rhinophores are retracted but just visible in most of the material. The white gills were only extended in two preserved specimens, the holotype SMF 360586 and SMF 360587, and the large gill cavity with its thin rim is clearly visible (Fig. 9B).

All preserved specimens are curled ventrally to a greater or lesser extent except the holotype SMF 360586. The black lines remain on the dorsum and are visible through the hyponotum in the holotype SMF 360586 (Fig. 9D). The foot is narrower than the dorsum, more than 1/2 to 2/3 the width of the dorsum in the less curled specimens (SMF 360586, SMF 360589). The penis is extruded in specimen A of SMF 360589 (Fig. 9E). The foot is rounded anteriorly and tapered posteriorly. The anterior margin is bilabiate and both edges appear to be clearly notched in specimen B of SMF 360589 (Fig. 9F) but this is an artefact of preservation. The oral tentacles are indistinct in all preserved specimens, certainly not as obvious as those of *P. liturata* or, in fact, most dorids. None of the photographs are helpful in showing them, although the bilabiate margins are just visible in Fig. 9E.

**Figure 10.**
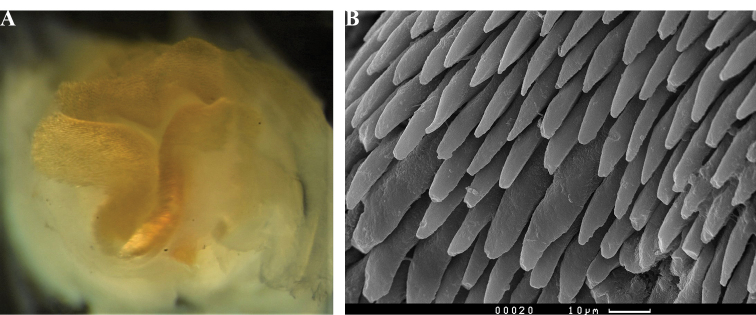
*Paradoris hypocrita* sp. nov., jaws of paratype SMF 360588 **A** light micrograph of three plates **B** SEM of rodlets.

**Figure 11.**
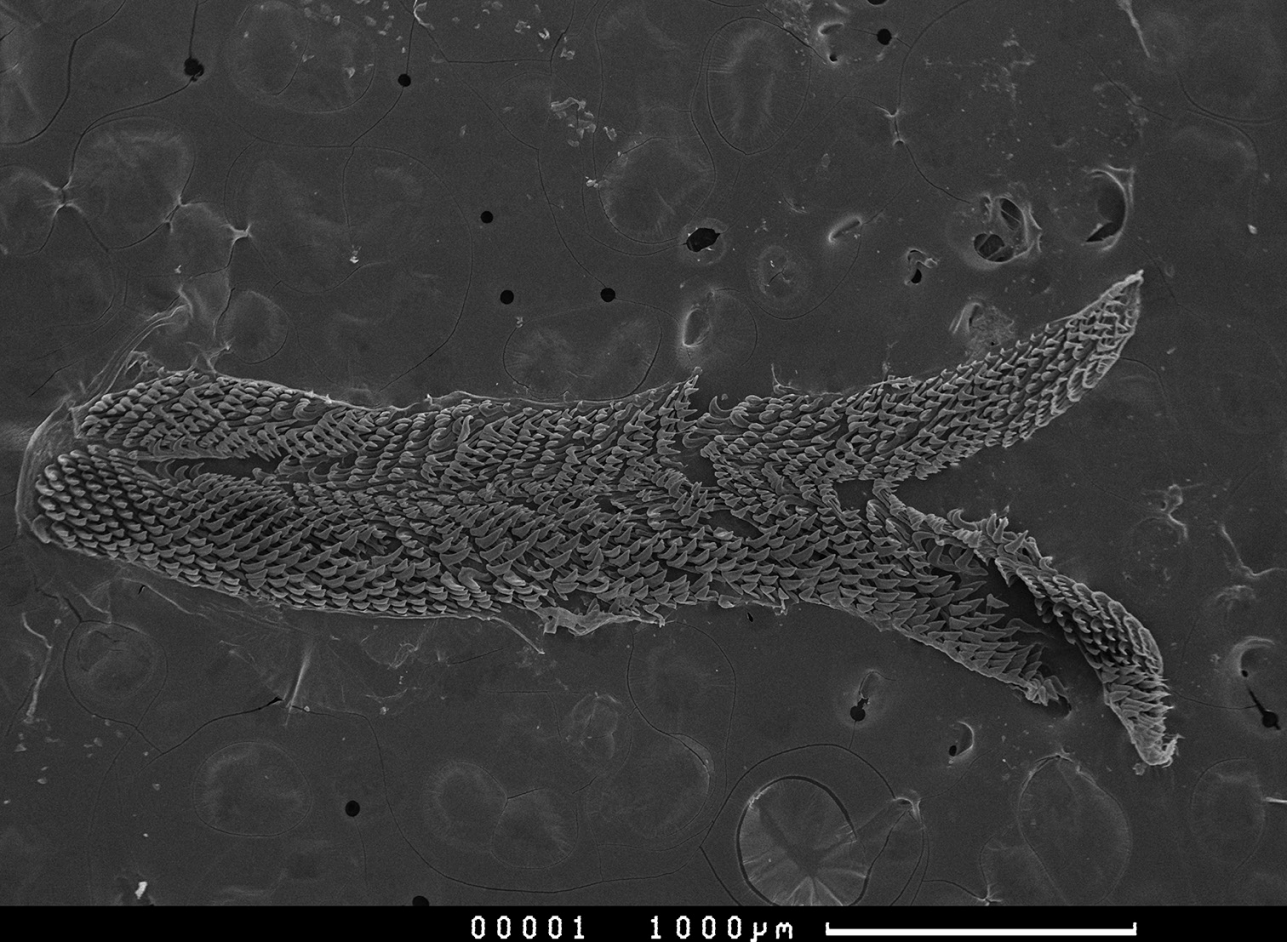
*Paradoris hypocrita* sp. nov., SEM of whole radula of paratype SMF 360588.

**Figure 12.**
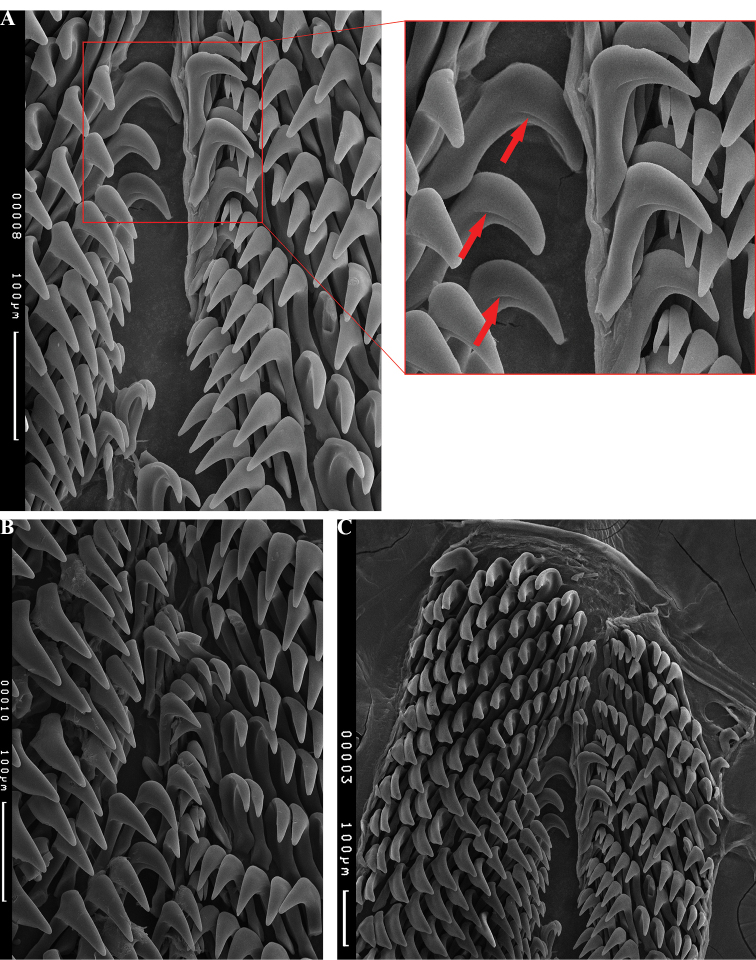
*Paradoris hypocrita* sp. nov., SEM radular images **A** middle rows [from Fig. 12C] magnified to show a few of the teeth with the distinctive groove (arrowed); paratype SMF 360588 **B** newer rows of paratype SMF 360589 specimen B) **C** old end of radula, paratype SMF 360588.

The jaws are formed of three plates (Fig. 10A). The rodlets are slightly curved, each with a tapered rounded tip; the more worn rodlets have a rounder tip, and some are broken off (Fig. 10B). The radula is asymmetrical and there are more teeth per row on the left side than the right side. The general shape of the radula is distinctive for the two *Phyllidiella* mimics, *P. liturata* and *P. hypocrita*: long and narrow, rounded at the old end, and with two long tails of sharp teeth at the new end (Fig. 11). The radular formula of *Paradoris hypocrita* sp. nov. (n = 2) is 53–55 × 14–16 (left).0.8–11 (right).There is no rachidian, but a narrow space is present down the middle of the radula in its place (Fig. 12B, C). The hooks of the lateral teeth are grooved, which is very difficult to see (Fig. 12A, arrowed).

###### Remarks.

This species appears to be relatively common in the northernmost part of the Red Sea, based on the available photographs (Rudman 2007a, except the photograph from Borneo). It differs consistently in external morphology from *Paradoris liturata*, which is currently recorded only from Indonesia and PNG (Dayrat 2006), Malaysia (Masayoshi 2002), and the Philippines (Okiedivenut 2007). Note that the Red Sea species is easily distinguished from the west Pacific species on iNaturalist (2007–2020) and that there are no records in the Indian Ocean. Discodorid species are known to vary in notum colour and pattern and rhinophore lamellae counts, but the following differences between *P. hypocrita* and *P. liturata* can be observed.

Externally, the black pattern of *P. hypocrita* sp. nov. forms a series of paired, loose polygons on the dorsum, sometimes incomplete, but in *P. liturata* the two or three black lines are longitudinal, either complete or broken (but note the black ground colour in a photograph (no specimen available) in Dayrat (2006: fig. 17) H from Papua New Guinea). The dorsum is (always) pink in *P. hypocrita* (described as grey in *P. liturata* (Dayrat 2006) but note the pink tinge in Dayrat (2006: fig. 17) and on Sea Slug Forum). There are 17 lamellae counted from photographs (specimens are contracted) in *P. hypocrita*, while *Paradoris liturata* has 13, 15, or 16 lamellae on the rhinophores. However, one head shot of *P. hypocrita* bears 13 or 14 lamellae on one side and 16 or 17 on the other, indicating similar variations in both species. There are 6–8 gills in *P. liturata* but only six in *P. hypocrita*, coloured various shades of grey in the first species and white in the latter.

The three jaw plates and form of the radulae and teeth are very similar if not identical in both species, albeit based on few specimens, but the numbers vary with fewer teeth per row in *P. hypocrita* sp. nov. The radular formula of *P. liturata* is 45–79 × 18–21 (left). 0.12–14 (right) (n = 4) while in *P. hypocrita* the formula is 53–55 × 14–16 (left). 0.8–11 (right) (n = 2). Tooth shape in discodorids is similar and at this level of magnification no particular differences are visible. It may be that Dayrat (2006) is correct and this species forms part of a very variable *P. liturata* species. However, the high endemism of nudibranchs in the Red Sea currently being revealed (see Discussion) combined with the consistent polygonal dorsal pattern and fewer teeth per row in the radula of the Red Sea specimens examined are considered sufficient to warrant separation. It is also noteworthy that there are no published records of *P. liturata* from the Indian Ocean: the westernmost Pacific record is from Indonesia (Dayrat 2006), further supporting the distinctiveness of the Red sea species.

*Paradoris liturata* has not been recorded in the Indian Ocean but there are at least two species of *Paradoris* resembling phyllidiids which remain unidentified in the western Indian Ocean. One is pink with three to many longitudinal, usually broken, lines, granular tubercles of different sizes, and grey gills (Bidgrain 2020a; Anderson 1988–2020); this may prove to be *P. liturata* and if so, would the first records of this western Pacific species in the Indian Ocean. The second species is white with five longitudinal wavy black lines and evenly sized tubercles and is probably a new species (Bidgrain 2020b). The Red Sea species clearly differs from both of these in the pattern of the black lines; additionally there are no records of either undescribed species in the Red Sea.

###### Etymology.

This epithet is based on the Latin noun *hypocrita* (mime, mimic) and refers to its superficial resemblance to another family, the Phyllidiidae.

### Check-list of Phyllidiidae recorded from the Red Sea, with distribution range

Phyllidia (Fryeria) rueppelii (Bergh, 1869) Red Sea, Gulf of Oman

*Phyllidia multifaria* Yonow, 1986 **Endemic**

*Phyllidia schupporum* Fahrner & Schrödl, 2000 Red Sea, Persian Gulf, Gulf of Oman

*Phyllidia* sp. [Eliot 1908 as *varicosa*] ??

*Phyllidia undula* Yonow, 1986 Red Sea, East Africa

*Phyllidia varicosa* Lamarck, 1801 Indo-West Pacific

*Phyllidiella amphitrite* sp. nov. **Endemic** (presumed)

*Phyllidiella* ‘*pustulosa*’ (Cuvier, 1804) Indo-West Pacific (currently)

‘*Phyllidia’ sudanensis* (Heller & Thompson, 1983) **Endemic** (or is it the western IO *P. meandrina*?)

*Phyllidiella zeylanica* (Kelaart, 1858) Western Indian Ocean

*Phyllidiopsis cardinalis* Bergh, 1873 Indo-West Pacific

*Phyllidiopsis dautzenbergi* (Vayssière, 1912) **Endemic**

*Phyllidiopsis monacha* (Yonow, 1986) **Endemic**

*Phyllidiopsis sinaiensis* (Yonow, 1986) **Endemic** (including Gulf of Tadjourah)

*Phyllidiopsis* sp. 1 **Endemic** (presumed)

*Phyllidiopsis* sp. 2 **Endemic** (presumed)

## Discussion

This work on the Red Sea phyllidiids brings the total number of species recorded in the family to 16 for the region. Of these, four named species are considered endemic: *Phyllidia multifaria*, *Phyllidiopsis dautzenbergi*, *Phyllidiopsis monacha*, and *Phyllidiopsis sinaiensis*. Most likely *Phyllidiella amphitrite* sp. nov. and ‘Phyllidia’ sudanensis are endemic, and if the two unnamed *Phyllidiopsis* are included, they would bring the total endemic phyllidiids to eight, a high percentage within the family. In light of recent papers designating more new nudibranch species as endemic to the Red Sea, percentages may well increase in other groups. Yonow (2018) described three new endemic chromodorid species, Epstein et al. (2018) described a further two endemic chromodorids, and Matsuda and Gosliner (2018) two more; Yonow (2008) had noted that *Chromodoris aspersa* from the Red Sea was different from the species recorded from the Indo-West Pacific and this was proved true: the Red Sea species was described as *C. baqe* Bonomi & Gosliner, 2020 and endemic. In two years, the endemic species of Chromodorididae was increased by eight species, increasing endemism to 27% and there remain more un-named species. *Coryphellina rubrolineata* O’Donoghue, 1929 has also been restricted to its type locality, the Red Sea, and differentiated from its West Pacific sibling species by Ekimova et al. (2020). They had no specimens from the Indian Ocean in their analysis: their “Arabian Gulf” specimen was collected in the Gulf of Tadjourah, at the mouth of the Red Sea; therefore, their comparison was actually between Red Sea specimens and western Pacific sequences obtained from GenBank.

Fahrner and Schrödl (2000a) redescribed *P. sinaiensis* and reviewed the species of the Red Sea. They followed the synonymies of Brunckhorst (1993), concluding that there were eleven species in the Red Sea of which four were endemic (36%). The same three species are still endemic, *P. dautzenbergi*, *P. sinaiensis*, and *P. schupporum*, but P. (F.) rueppelii is no longer considered endemic (see below). They identified *P. multifaria* as the West Pacific *P. elegans* but *P. multifaria* is a valid and endemic species. *Phyllidia sudanensis* was identified as *P. annulata* and *P. undula* as *P. ocellata* in their paper.

**Plate 19.**
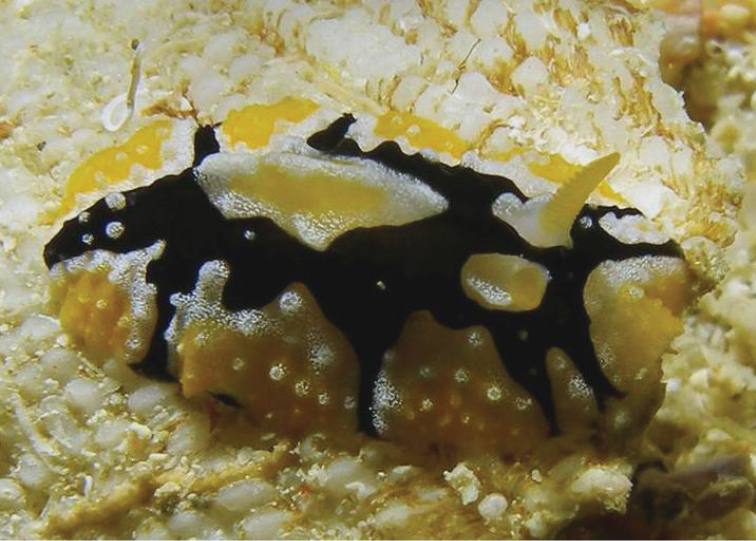
*Phyllidiopsis monacha*, Hurghada, 13 Dec 2014 (photograph only S. Kahlbrock).

No specimens of *P. monacha* or *P. sudanensis* were found again for further examination. *Phyllidiopsis monacha* is recorded by two recent high-resolution images of one individual (Plate 19), so its identity is substantiated, and the detailed original description is recognisable (despite comments by Rudman (2007b); if individual specimens are carefully and clearly described and illustrated, individually, they can be recognised again (also advocated by Yonow and Jensen 2018: 35, 42). The individuals recorded as *P. monacha* from Indonesia by Gosliner et al. (2008) and Rutland Island (Andaman and Nicobar Islands) by Raghunathan (2015) are considered a different species, having a central yellow or orange dorsum followed by concentric bands of white, black, and white, followed by an orange or yellow skirt.

The identity of *P. sudanensis* remains a mystery (12 mm pres.; Suakin, Sudan). There is no holotype and no photographs of the living specimen and the serial section slides of it are lost (Prof. JA Heller, Hebrew University of Jerusalem, pers. comm.). Copies of the original colour slides made of the Suakin Expedition were obtained courtesy of Prof. CD Todd (University of St. Andrews) and no images were present of the living *P. sudanensis*. The poor description of *P. sudanensis* means that it cannot be assigned to a genus, but probably belongs to *Phyllidiella*. Fahrner and Schrödl (2000a) assigned this specimen to *P. annulata*, a West Pacific species, presumably based on Brunckhorst’s (1993) synonymy which also included *P. meandrina*. As Rudman (2002) noted, *P. sudanensis* was more likely to be *P. meandrina* Pruvot-Fol (1957, type locality Mauritius). *Phyllidiella meandrina* was removed from synonymy with *P. annulata* when the syntype was examined by Yonow et al. (2002: 864), who provided a thorough comparison and clear separation of the two species. *Phyllidiella meandrina* is relatively common in the western Indian Ocean but there are no records of it from the Red Sea. While there is some resemblance, the synonymy of *P. sudanensis* with *P. meandrina* does not seem warranted at present.

A second species also remains a mystery, undiscovered since its old record: it was described by Eliot (1908) from Sudan as *Phyllidia varicosa*, citing the notes provided on the living sea slug:

*On sand among coral at the edge of the shore-reef*; *seen at a depth of about a fathom and obtained by a diver. 6 cm long and 3 cm broad. Jet black with raised warts of a dirty greenish white*, *which are very high and bear small secondary warts*; *the tops of these are brilliant orange. The rhinophores are also orange and were kept retracted though the animal was continually crawling. The largest warts are arranged one behind the other in five longitudinal rows down the back. From the outermost of these rows low bands of greenish grey bearing small warts go to the mantle-edge.*

Eliot then goes on to say that the orange tips were harder than the remaining epidermis. Brunckhorst (1993) included this species in *P. varicosa*; Fahrner and Schrödl (2000a) decided to discount the orange colouration (and presumably the orange rhinophores) and identified this record as *P. rosans*. Certainly, a black and white drawing of this description [and the drawing of the record by Quoy and Gaimard (1832) from New Ireland, Papua New Guinea as *P. trilineata* cited in Yonow (1986, 1988)] are very similar to *P. rosans*, which is very broadly oval with a central ridge and two lateral tuberculate ridges on each side (not one as in *P. varicosa*), followed by one or two rings of tubercles, and a tuberculate mantle margin. Despite intensive diving and photography in the Red Sea in recent years, nothing has been photographed or collected resembling this species. *Phyllidiella rosans* in the western Indian Ocean (Yonow et al. 2002; Yonow 2012) may be different from the Pacific species (type locality Tahiti) as they differ considerably (except *P. soria* Marcus & Marcus, 1970; type locality also Tahiti): Gosliner et al. (2008) only record *P. rosans* from the western Indian Ocean and the mid-Pacific, stating that “there may actually be two species one in the Indian Ocean and a second one in the central Pacific.”

**Plate 20.**
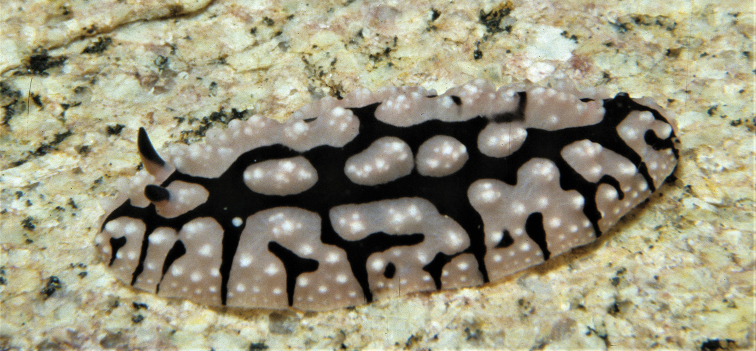
*Phyllidiopsis sinaiensis*, Sha’arm el Sheikh, 24 Dec 1990 (N. Yonow, NY #111).

The final species in the check-list needing comment are two *Phyllidiopsis* species, which have not been collected but have each been photographed several times. The first (lower left in Yonow 2008: 275) differs from *P. sinaiensis* (Plate 20) in having high and rounded simpler tubercles, and the black area is ‘painted’ in a different pattern. It is possible that the species illustrated in Yonow (2015: 541) may be a larger, more tuberculate *P. sinaiensis* with a preponderance of black, but without material the identities of these remain unknown. *Phyllidiopsis sinaiensis* is another species in which the black lines can be very thin or much heavier (e.g., Blatterer 2019: pl. 195): currently they are all identified as *P. sinaiensis*.

Of the non-endemic species, *Phyllidia schupporum* is recorded from the Persian Gulf for the first time in this work: Nithyanandan (2012) recorded it as P. (F.) rueppelii, but it is re-identified from the original photographs; several additional photographs made available from the United Arab Emirates also pertain to this species (Carole Harris, Sydney, Australia, pers. comm.) and there are a few misidentified as *rueppelii* on the internet. The other species which had been considered endemic to the Red Sea but is also found in the UAE is P. (F.) rueppelii (Carole Harris, Sydney, Australia, pers. comm.). As a result, these two species are no longer considered endemic to the Red Sea. It is worth noting that Rezai et al. (2016) recorded *Phyllidia rueppelii* in a check-list of species from the Persian Gulf, but there is no description or photograph.

**Plate 21.**
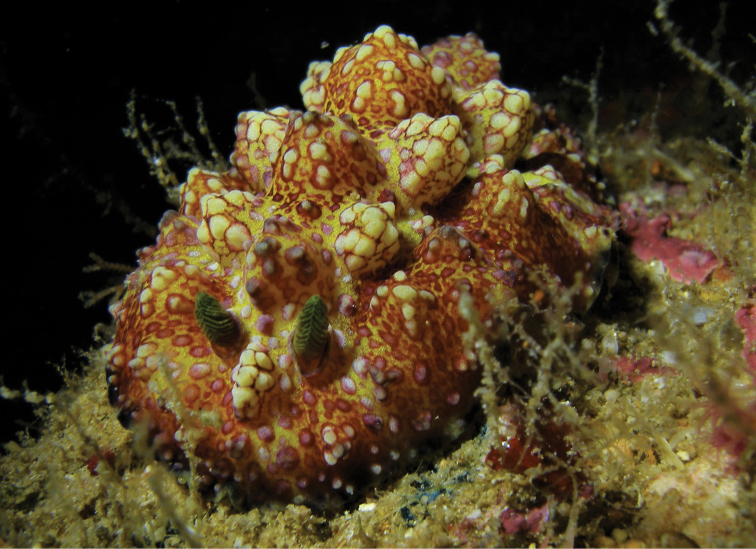
*Phyllidiopsis cardinalis*, Hurghada, 15 July 2010 (photograph only, S. Kahlbrock).

Two species from the Red Sea have more extended western Indian Ocean distributions: *Phyllidia undula*, part of the *P. multituberculata* complex, is found south along the east African coastline, and *Phyllidiella zeylanica* is recorded as far east as southwest Thailand (Yonow 1996) and Papua New Guinea (Domínguez et al. 2007). Three species, *Phyllidiopsis cardinalis*, *Phyllidiella* ‘*pustulosa*’, and *Phyllidia varicosa* have much wider Indo-West Pacific distributions. *Phyllidiopsis cardinalis* has not been collected from the Red Sea, but there are numerous photographs of it in the author’s archive (Plate 21) indicating that it may be moderately common in the very north of the Red Sea. One of the first published photographic records can be found in Debelius (1998: 225) and most recently in Blatterer (2019: plate 195). It is clearly recognisable, with its very complicated dorsal colouration and equally complex tubercular arrangement, and currently has a wide Indo-Pacific distribution, from the Red Sea through the Indian Ocean across the Pacific to Hawai’i.

The most photographed species in the Red Sea, and therefore presumed to be the most common, are *Phyllidia multifaria*, *Phyllidia undula*, *Phyllidia varicosa*, Phyllidia (Fryeria) rueppelii, *Phyllidiopsis sinaiensis*, and *Phyllidiella* ‘*pustulosa*’. Of these, only *P. sinaiensis* is endemic, *P. undula* and P. (F.) rueppelii are restricted to the western Indian Ocean, and *P. ‘pustulosa*’ (currently) and *P. varicosa* have widespread ranges in the Indo-Pacific.

## Supplementary Material

XML Treatment for Phyllidiella amphitrite

XML Treatment for Phyllidiella zeylanica

XML Treatment for Phyllidia schupporum

XML Treatment for Paradoris hypocrita

## Figures and Tables

**Figure A1.**
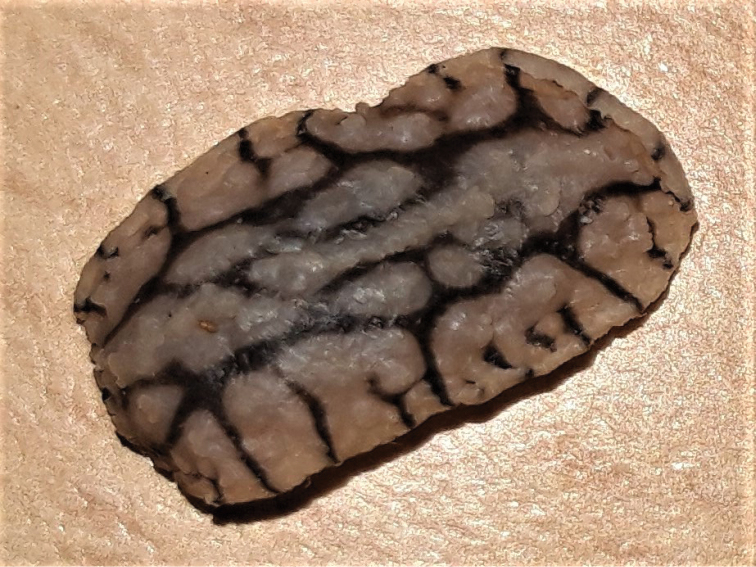
*Phyllidiopsis sinaiensis*, photograph of preserved specimen collected by the ‘Meteor’ expedition at 214–237 meters depth. Anus visible to the right of the photograph, at the narrowing of the longitudinal black lines.

## Appendix 1

Additional specimens of species previously described from the Red Sea (Yonow 1986, 1988, 1996)

***Phyllidia multifaria* Yonow, 1986**

Small Gubal Island, Hurghada, Egypt, 10 Sept 2012, 2–5 m depth, 15 mm alive (13 mm pres., alcohol), leg. S. Kahlbrock.

TQ 1, Sanganeb Reef, Sudan, 29 Mar 1991, 4.5 m depth, 25 × 14 mm pres., formalin, leg. T. Paulus (ref # 30).

**Diagnosis.** Common, endemic, up to 45 mm. Black, white, and orange with four longitudinal black lines parallel to three rows of orange-tipped tubercles. The outer line on each side extends to margin in rays creating scallops. Black line on sole of foot.

**Similar species.***Phyllidia elegans* Bergh, 1869 (eastern Indian Ocean and West Pacific).

***Phyllidiella ‘pustulosa*’ (Cuvier, 1804)**

Lighthouse, south of Port Sudan harbour, Sudan, 18 Mar 1991, 11 m depth, 25 × 10 mm pres. alcohol, leg. and photographs T. Paulus.

Small Gubal Island, Hurghada, Egypt, Oct 2012, one spcm 18 × 8.5 mm pres., formalin, leg. S. Kahlbrock.

**Diagnosis.** Common, up to 70 mm in length. Pink tubercular areas arranged in five or six clusters on the midline, one ring of lateral clusters, pink margin. The black pigment can be thick or thin between tubercular groups. Tubercles may be ‘spiky’ (Plate 3). No black line on sole of foot.

**Remarks.***Phyllidiella* ‘*pustulosa*’ is a common species found throughout the Indo-West Pacific and as such is variable: Stoffels et al. (2016) indicated that there are at least four clades in the *P. pustulosa* complex in the western Pacific; however, as no Red Sea or Indian Ocean specimens were included in their analysis, there are almost certainly more cryptic species to be revealed. More recently, Bogdanov et al. (2020) analysed the chemical components of 52 Indonesian specimens and found that, amazingly, there was a correlation of specific secondary metabolite profiles with their concurrent molecular phylogeny of seven clades. Therefore, it is likely that the Red Sea species of *P.* ‘*pustulosa*’ is a separate species, in which case the name *P. melanocera* (Yonow, 1986) would apply, since it is the only new name based solely and specifically on Red Sea material, albeit a juvenile individual.

***Phyllidiopsis dautzenbergi* (Vayssière, 1912)**

Hurghada, Egypt, May–Sept 2010, one specimen 9 × 4 mm curled (pres., formalin), leg. S. Kahlbrock.

Makadi Bay, Egypt, 3 Aug 2013, photograph Hsini Lin.

**Diagnosis.** Moderately rare, endemic, 20 mm. Black and white, very constant dorsal pattern, white rhinophores. Black lines form ellipse around median white ridge on the dorsum with five paired black lines extending from it to the margin. Tiny pustules in the white areas. No black line on sole of foot. These are the first and most northerly records in the Red.

**Similar species.** None.

***Phyllidiopsis monacha* (Yonow, 1986)**

Tobia Arba, Safaga, Egypt, 13 Dec 2014, two photographs of one individual matching holotype description perfectly, S. Kahlbrock (Plate 18).

**Diagnosis.** Rare, endemic, 20 mm, black, white, and orange (or yellow) dorsal pattern. Central dorsum white and orange (or yellow) with median tuberculate ridge surrounded by black; two coloured patches around the yellow (or orange) rhinophores. Variably sized tubercles on the black, white, and coloured areas. Thick black pattern is variable and ’messy’ compared to that of *P. dautzenbergi*.

**Remarks.** Red Sea endemic, this small species is clearly very rare, with only two individuals photographed since its original description (one individual illustrated in Yonow 2008).

**Similar species.**Phyllidiopsis cf. monacha (Indo-West Pacific).

***Phyllidiopsis sinaiensis* (Yonow, 1988)**

Bab el Mandab, Red Sea, ‘Meteor’ cruise 5, stn. 230 KD2, 05 Mar 1987, 214–237 m depth, 18 × 11 mm pres., SMF collections (no reg. no.; alcohol; Fig. A1). This small specimen still retains the black markings as well as very faint black-tipped oral tentacles when examined in 2000; the black on the dorsum was still defined when examined in 2020. The depth at which this specimen was found is noteworthy.

Near Gardens, Sha’arm el Sheikh, Egypt, 24 Dec 1990, 18 m depth, 48 × 18 mm alive; 35 × 16 mm pres., leg. and photographs N. Yonow (ref Sha’arm NY #111; formaldehyde; Plate 19). My notes on the live material stated that the specimen was incredibly smelly, and that the oral tentacles were tipped with black; this black pigmentation had disappeared in preservative (formalin) and was greenish in colour in 2000.

Dahab, Egypt, Nov 1990, 43 × 20 mm pres., leg. and photographs J.P. Hausmann (no black pigment remaining in 2000, formaldehyde).

Sanganeb Reef, Sudan, 29 Mar 1991, 45 × 27 mm pres., leg. T. Paulus (ref # 38) (black-tipped oral tentacles upon reception of specimen, formaldehyde; no black pigment remaining in 2000).

Jeddah, Saudi Arabia, 1980s, photograph of one individual, W. Pridgen.

Hurghada, Egypt, 2010, photographs of four individuals, S. Kahlbrock.

Eilat, Israel, 2019, photographs of one individual, R. Amar.

**Diagnosis.** Common, largest of the Red Sea phyllidiids, up to 85 mm, black markings and pink/white complex tubercles on the dorsum. Complicated black pattern can comprise variably thick or thin lines. Two main black lines start as one at frontal margin and divide after rhinophores, meeting behind anal papilla with many irregular and sub-dividing lines to margin along the front, sides, and back. In larger specimens, second pair of meandering black lines either side of central row of complicated and compound tubercles. Second row of complex central tubercles present on either side, and compound tubercles in white/pink spaces created by black pattern. Rhinophores bicoloured black and pink; fused oral tentacles tipped in black; no black line on the sole.

**Remarks.** It is unfortunate that neither Brunckhorst (1993) nor Fahrner and Schrödl (2000) recognised Phyllidia ? varicosa var. quadrilineata in Yonow (1986: figs 7, 8, 12E) as a typical *P. sinaiensis*.

**Similar species.***Phyllidiopsis krempfi* Pruvot-Fol, 1957 (Indo-West Pacific). *Phyllidiopsis fissuratus* Brunckhorst, 1993 (western Pacific).

## Appendix 2

**Additional material from the Farasan Banks southern Red Sea.** This semi-submerged coral reef system is located almost opposite Sanganeb Reef, near the Saudi Arabian coastline, and is approximately 560 km long and 50 km wide (Map 1). The specimens were collected by a KAUST expedition between in May 2017 and are lodged in the mollusc collection of Naturalis. Each specimen was examined under a binocular microscope; they had not been relaxed so were not flat (preserved in alcohol).

***Phyllidia multifaria* Yonow, 1986**: three specimens 30–40 mm long, preserved but bent or folded.

***Phyllidia varicosa* Lamarck, 1801**: one specimen 55 mm in length, preserved.

***Phyllidiella ‘pustulosa*’ (Cuvier, 1804)**: 21 specimens 14–48 mm, preserved; most are folded or bent, and some specimens had dried out.
