# Corrigendum: Red Sea Opisthobranchia 6: Phyllidiidae and their paradorid mimic: new species and new records (Heterobranchia, Nudibranchia, Doridina). ZooKeys 1006: 1–34. https://doi.org/10.3897/zookeys.1006.59732

**DOI:** 10.3897/zookeys.1055.72258

**Published:** 2021-08-12

**Authors:** Nathalie Yonow

**Affiliations:** 1 Department of Biosciences, Swansea University, Singleton Park, Swansea SA2 8PP, Wales, United Kingdom Swansea University Swansea United Kingdom

## Abstract

none

The paper was written during lockdown 2019–2020, during which I could not access the type of *Phyllidiamonacha* Yonow, 1986 at the Natural History Museum in London (NHMUK) to confirm its generic status. In the first modern revision of phyllidiid genera, Brunckhorst (1993: 64) stated that he examined the holotypes of both *P.monacha* Yonow, 1986 and *Phyllidiadautzenbergi* Vayssière, 1912 and that they were the same species; he therefore assigned both of them to the genus *Phyllidiopsis* Bergh, 1876, which has fused oral tentacles. No new specimens of *P.monacha* have ever been recovered for reinvestigation and in that paper it was listed as a separate species with new photographs, but retained in *Phyllidiopsis*.

In fact, the original description (Yonow 1986: 1408) clearly states that “The tentacles are simple conical structures” and the drawing also shows two separated triangular oral tentacles and a divided anterior foot margin (Yonow 1986: fig. 3B). These oral tentacles are not diagnostic of *Phyllidiopsis*, which has fused oral tentacles into a single unit. Images of the holotype of *P.monacha* (Figures 1, 3, 5) and the specimens of *P.dautzenbergi* (Figures 2, 4, 6) have just been obtained from the NHMUK, and confirm that the original designation of *Phyllidiamonacha* was correct. These images of both species (photographs of the three specimens of *P.dautzenbergi* were provided) confirm that they are not the same species and that they do not belong in the same genus. Therefore, all references in the paper to *Phyllidiopsismonacha* should be corrected to *Phyllidiamonacha*: page 2, Introduction; page 23, Check-list and Discussion; page 24, Discussion and Plate 19; and page 32, Appendix 1.

**Figures 1–6.**
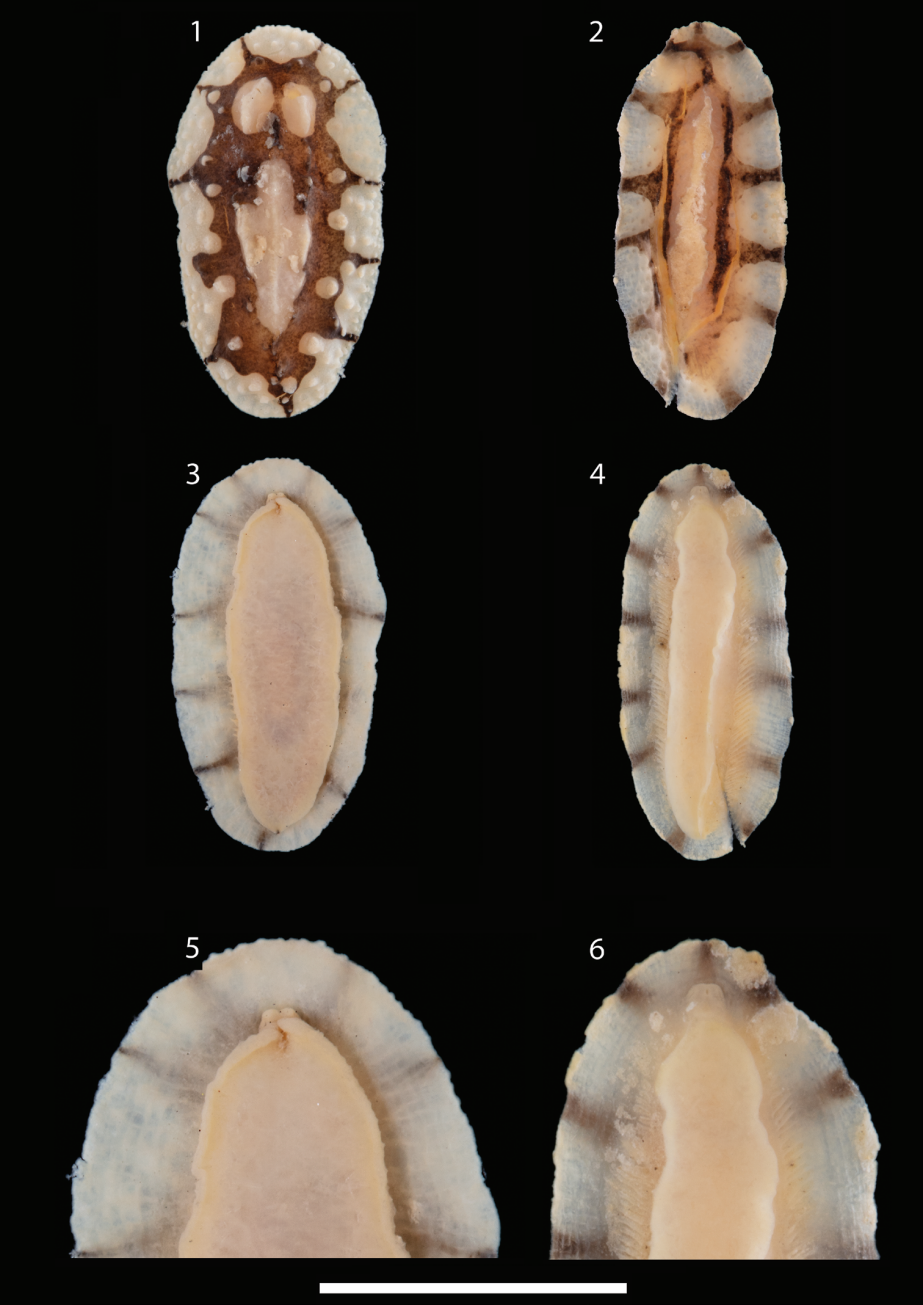
**1, 3, 5** holotype of *Phyllidiamonacha*, NHMUK 1985205 **2, 4, 6** specimen of *Phyllidiopsisdautzenbergi*, NHMUK 20210058, illustrated here as it was not figured by Yonow (1986). This is also the specimen that was dissected by Brunckhorst (1993) but clearly he did not dissect *Phyllidiamonacha*. **1, 2** dorsal views **3, 4** ventral views **5, 6** close-up views of the anterior foot margins. Scale bar: 5 mm (**1–4)**; 10 mm (**5, 6)**.

For completeness, the four relevant specimens from Yonow (1986), with their current museum catalogue numbers, are listed below:

- Phyllidia dautzenbergi Vayssière, 1912, 1 specimen 19 × 5 mm, NHMUK 20210058, Jezirat Seba, Djibouti, Red Sea, 10–15 m depth, 23 June 1983.
- Phyllidia dautzenbergi Vayssière, 1912, 1 specimen 6 × 3 mm (preserved), NHMUK 20210059, South Tower reef, Saudi Arabia, Red Sea, 15 m depth, 16 March 1984.
- Phyllidia dautzenbergi Vayssière, 1912, 1 specimen 6 × 4 mm, NHMUK 20210060, Sha’ab Rumi, Sudan, Red Sea, 10–15 m depth, 6 July 1983.
- Phyllidia monacha Yonow, 1986, holotype 14 × 7.5 mm, NHMUK 1985205, Creek, Jeddah, Saudi Arabia, Red Sea, 8 m depth, 15 December 1983.
